# γ-Glutamyltranspeptidase is an endogenous activator of Toll-like receptor 4-mediated osteoclastogenesis

**DOI:** 10.1038/srep35930

**Published:** 2016-10-24

**Authors:** Sawako Moriwaki, Takeshi Into, Keiko Suzuki, Mutsumi Miyauchi, Takashi Takata, Keigo Shibayama, Shumpei Niida

**Affiliations:** 1 Biobank, Medical Genome Center, National Center for Geriatrics and Gerontology, Obu 474-8511, Japan; 2 Department of Oral Microbiology, Division of Oral Infections and Health Sciences, Asahi University School of Dentistry, Mizuho 501-0296, Japan; 3 Department of Pharmacology, School of Dentistry, Showa University, Tokyo 142-8555, Japan; 4 Department of Oral and Maxillofacial Pathology, Institute of Biomedical & Health Sciences, Hiroshima University, Hiroshima 734-8522, Japan; 5 Department of Bacteriology II, National Institute of Infectious Diseases, Tokyo 208-0011, Japan

## Abstract

Chronic inflammation-associated bone destruction, which is observed in rheumatoid arthritis (RA) and periodontitis, is mediated by excessive osteoclastogenesis. We showed previously that γ-glutamyltranspeptidase (GGT), an enzyme involved in glutathione metabolism, acts as an endogenous activator of such pathological osteoclastogenesis, independent of its enzymatic activity. GGT accumulation is clinically observed in the joints of RA patients, and, in animals, the administration of recombinant GGT to the gingival sulcus as an *in vivo* periodontitis model induces an increase in the number of osteoclasts. However, the underlying mechanisms of this process remain unclear. Here, we report that Toll-like receptor 4 (TLR4) recognizes GGT to activate inflammation-associated osteoclastogenesis. Unlike lipopolysaccharide, GGT is sensitive to proteinase K treatment and insensitive to polymyxin B treatment. TLR4 deficiency abrogates GGT-induced osteoclastogenesis and activation of NF-κB and MAPK signaling in precursor cells. Additionally, GGT does not induce osteoclastogenesis in cells lacking the signaling adaptor MyD88. The administration of GGT to the gingival sulcus induces increased osteoclastogenesis in wild-type mice, but does not induce it in TLR4-deficient mice. Our findings elucidate a novel mechanism of inflammation-associated osteoclastogenesis, which involves TLR4 recognition of GGT and subsequent activation of MyD88-dependent signaling.

Inflammation represents a protective immunovascular response of tissues to harmful stimuli; however, when not strictly controlled, it may lead to chronic persistent inflammation^1^. During the initial phase of chronic inflammation, cytokines or some endogenous activators of the innate immunity are thought to provide booster signals for injurious immune responses that influence the progress of diseases^2^,^3^,^4^. In the case of tissues close to the bone, chronic inflammation causes bone destruction, which is observed in disorders such as rheumatoid arthritis (RA), periodontitis, prosthetic loosening, and peri-implantitis^5^,^6^,^7^. Bone destruction is mediated by excessive formation of osteoclasts, which pathologically degrade the bone before the formation of the new bone^8^. The differentiation of these cells is thought to be mediated through an excessive production of osteoclastogenic factors, including receptor activator of nuclear factor kappa-B ligand (RANKL), which can be induced by inflammatory cytokines, including tumor necrosis factor (TNF)-α and interleukin (IL)-6, and also by the endogenous activators of innate immunity^5^,^8^. However, the precise mechanisms of chronic inflammation-associated osteoclastogenesis are not fully understood.

Innate immune responses can be activated by microbial products called pathogen-associated molecular patterns (PAMPs)^9^. PAMPs are recognized through the several classes of germline-encoded pattern recognition receptors (PRRs), including Toll-like receptors (TLRs), which can trigger proinflammatory protective immune responses against pathogens^10^. TLR4 represents one of the best-characterized PRRs and it serves as a receptor for bacterial lipopolysaccharide (LPS). In addition, PRRs are able to recognize certain endogenous molecules called damage-associated molecular patterns (DAMPs)^11^, which are closely associated with the induction and development of sterile inflammation observed in the diseases such as autoimmune diseases, Alzheimer’s disease, myocardial infarction, and atherosclerosis^12^. DAMPs usually emerge after tissue stress or injury, but, in physiologically normal circumstances, they can not be recognized by PRRs because of the intracellular compartmentalization or sequestration within the extracellular matrix. TLR4 can serve as a receptor for several kinds of DAMPs, including S100A8/S100A9 proteins, syndecans, and heparan sulfate, and can induce proinflammatory reactions^11^,^13^. TLR4 also recognizes tenascin-C, a component of the extracellular matrix, leading to the induction of an autocrine loop for persistent inflammation^14^. DAMPs are thought to enhance the inflammatory reactions; therefore their concentration in the inflamed lesions may affect the outcome of the inflammation.

Previously, we investigated lymphocyte-derived endogenous osteoclastogenic factors, and identified γ-glutamyltranspeptidase (GGT; encoded by *Ggt1*) from a cDNA library of 630,000 candidates obtained from a murine T lymphocytic cell line using an *in vitro* expression cloning method^15^. GGT is an enzyme involved in the breakdown of extracellular glutathione into its constituent amino acids and the transfer of γ-glutamyl moiety to acceptor molecules^16^,^17^ and can control the metabolism of glutathione and cysteine in order to mitigate oxidative stress or inflammation^18^. We further demonstrated that the *Ggt1* transgenic mice with selective overexpression in bone marrow stromal cells systemically exhibit osteoporosis due to excessive osteoclastogenesis and bone resorption^19^, suggesting a preferential implication of GGT in bone metabolism. Additionally, we showed that enzyme-inactivated purified GGT isolated from the rat liver has osteoclastogenic activity and that anti-GGT polyclonal antibody, which does not affect the enzymatic activity of GGT, can attenuate the osteoclastogenic activity of GGT^15^. These findings indicate an unknown mechanism of GGT activity, which is not related to its enzymatic activity.

GGT is produced as an ectoenzyme, localized at the surface of various types of cells, but it can also be found in body fluids, including serum^20^. Even though it has not been determined how GGT is released extracellularly, previous studies have demonstrated that the production of both types of GGT can be elevated in response to oxidative stress^21^,^22^. Furthermore, increased levels of humoral GGT are known to be a clinical biomarker for injuries in the liver, gallbladder and biliary tract^18^, suggesting that injured cells in the damaged tissues are the source of extracellular GGT. Our previous study showed that GGT levels are elevated in the synovial fluids of RA patients, apparently because of the GGT originating from the lymphocytes or inflammatory cells accumulated in the lesions^23^. It was additionally found that *in vitro* osteoclastogenesis can be induced by GGT purified from the rat liver^15^ or recombinant GGT obtained using a baculoviral system^23^. In rats, the administration of recombinant GGT to the gingival sulcus induces an increase in the number of osteoclasts at the surface of the alveolar bone^23^. Moreover, in the collagen-induced arthritis mouse model, the administration of monoclonal antibody against GGT to the arthritic joints attenuates bone destruction through suppression of excessive osteoclast formation that is associated with inflammatory reactions^23^. These observations indicate that extracellularly released GGT possesses osteoclastogenic activity, but the underlying mechanisms have not been elucidated.

As described above, recent reports have demonstrated that TLRs recognize DAMPs as cognate ligands^11^,^13^,^14^. In this study, we therefore aimed to examine whether extracellularly released GGT can be recognized by TLRs, especially TLR4, to exert its osteoclastogenic activity. Using recombinant human GGT1 protein (rGGT), we show that the osteoclastogenic activity of GGT is not observed in TLR4-deficient cells. Additionally, similarly to TLR4-stimulating DAMPs or PAMPs, GGT could stimulate macrophages to induce the production of proinflammatory cytokines and type I interferon (IFN). Our findings propose an important mechanism of the recognition of extracellular GGT by TLR4, which may be involved in the development of inflammatory diseases accompanied with excessive osteoclastogenesis and bone destruction.

## Results

### Extracellular GGT stimulates osteoclastogenesis

**Figure 1:**
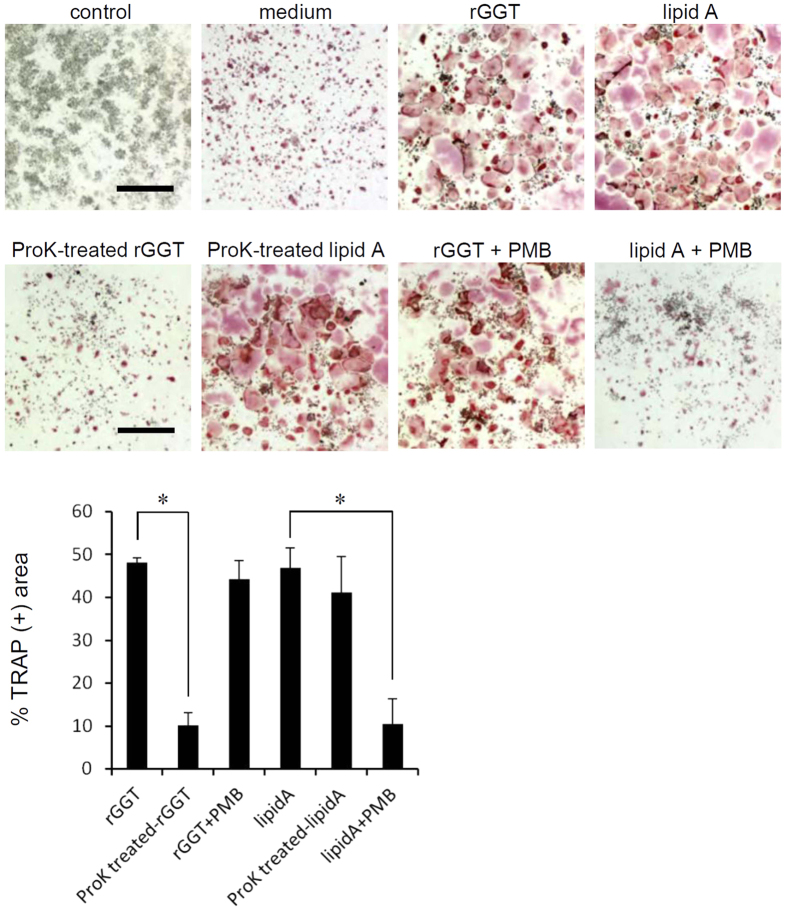
Extracellular GGT-induced osteoclastogenesis does not depend on LPS. RAW264.7 cells were differentiated into preosteoclasts by treatment with RANKL^low^ (20 ng/ml RANKL; panel of control). Preosteoclasts were stimulated with rGGT (200 ng/ml; panel of rGGT) or lipid A (50 ng/ml; panel of lipid A) in the absence of RANKL, or were left untreated (panel of medium) for 2 days. The effect of proteinase K pretreatment (panels of ProK-treated rGGT and ProK-treated lipid A) or PMB pretreatment (panels of rGGT + PMB and lipid A + PMB) on the activity of rGGT or lipid A was also tested. Cells were fixed and stained with TRAP. The percentage of the area of TRAP-positive cells was assessed from the microscopic images using the ImageJ software. Data are presented as the mean ± SD (n = 4). Scale: 200 μm. *p < 0.01.

First, to verify the osteoclastogenic activity of extracellular GGT, we performed an *in vitro* osteoclast-formation assay. Highly purified rGGT was prepared using a baculovirus system, as described previously^23^, and used as extracellular GGT for stimulation. Mouse primary mononuclear osteoclast precursors (OCPs) were generated by treatment of bone marrow hematopoietic cells with macrophage colony-stimulating factor (M-CSF). These OCPs were then treated with suboptimal doses of RANKL (RANKL^low^) to generate mononuclear tartrate-resistant acid phosphatase (TRAP)-positive committed OCPs (hereafter called preosteoclasts). Here we show that preosteoclasts were differentiated into multinucleated TRAP-positive osteoclast-like cells (OCLs) after stimulation with rGGT even in the absence of RANKL (Supplementary Fig. 1). In rGGT-stimulated OCPs, expression of the osteoclastic genes cathepsin K (*Ctsk*), matrix metallopeptidase 9 (*Mmp9*), c-fos (*Fos*) and NFATc1 (*Nfatc1*) were upregulated (Supplementary Fig. 2). Furthermore, the activity of rGGT was verified in human cells to be able to differentiate preosteoclasts into OCLs even in the absence of RANKL (Supplementary Fig. 3). The osteoclastogenic activity of rGGT was also tested using mouse macrophage RAW264.7 cells that do not require pretreatment with M-CSF. RAW264.7 cells were stimulated with RANKL^low^ to obtain preosteoclasts, and these cells were subsequently stimulated with rGGT in the absence of RANKL. We show that a number of TRAP-positive OCLs were generated by rGGT stimulation (Fig. 1). Immunocytochemical analysis of rGGT-induced OCLs showed that these cells produce a large quantity of the osteoclast marker cathepsin K (Supplementary Fig. 4). rGGT-induced OCLs exhibited bone resorption-like activity, which was assessed using an *in vitro* pit formation assay with calcified matrix-coated disks that are often used to assess the osteoclast-like functions (Supplementary Fig. 5). These results suggest that extracellular GGT can mediate the process of maturation of osteoclastogenesis although the presence of RANKL is essentially required for the process of initiation of osteoclastogenesis.

### Extracellular GGT stimulates osteoclastogenesis without LPS signaling

Bacterial LPS contamination during the preparation of recombinant proteins often leads to erroneous experimental results^24^. Although rGGT used in our experiments had been prepared without the use of bacterial cells, we tried to ensure that it is not contaminated. In OCPs, LPS is known to stimulate osteoclastogenesis^25^,^26^, and we show that the synthetic product of lipid A, an active site of LPS, indeed induced osteoclastogenesis in RAW264.7-derived preosteoclasts (Fig. 1). The osteoclastogenic activity of rGGT in preosteoclasts was tested after digestion with proteinase K. It was found that this treatment completely abrogated the activity of rGGT, while the activity of lipid A was not affected (Fig. 1). Treatment of rGGT with the antibiotic polymyxin B (PMB), a potent inhibitor of lipid A^27^, was also performed for the treatment of the preosteoclasts, and it was demonstrated that PMB did not affect rGGT-dependent OCL formation, while it significantly attenuated the activity of lipid A (Fig. 1). Furthermore, a *Limulus* amoebocyte lysate assay revealed that the amount of LPS included in our preparation of 1 mg/ml rGGT was at least 80 ng/ml or less. In our experiments, the prepared rGGT was diluted more than 4,000 times, resulting in LPS concentrations of less than 20 pg/ml in the cell culture. Concentrations of LPS less than 10 ng/ml did not affect osteoclastogenesis in RAW264.7-derived preosteoclasts (Supplementary Fig. 6).

It was additionally determined that rGGT-induced osteoclastogenesis in RAW264.7-derived preosteoclasts was not affected by the presence of osteoprotegerin, a decoy receptor for RANKL, or by the presence of anti-TNF-α neutralizing antibody (Supplementary Fig. 7). The antibody was effective as it could evidently block TNF-α activity in *Il1b* induction assay in RAW264.7 cells (Supplementary Fig. 8). These results suggest that the extracellular GGT-mediated stimulation of osteoclastogenesis is not dependent on the autocrine stimulation with RANKL and TNF-α. Furthermore, the analysis of localization using fluorescence-labeled rGGT showed that GGT was present at the surface of as well as inside OCPs (Supplementary Fig. 9). These observations suggest that extracellular GGT directly stimulates cells to activate osteoclastogenesis.

### GGT activates macrophages

Bone marrow-derived macrophages (BMDMs) were stimulated with rGGT, and the mRNA expression of proinflammatory cytokine and chemokine was investigated. The incubation times used in these experiments were shorter than those used for OCP stimulation. rGGT stimulation was shown to upregulate the mRNA expression of TNF-α (*Tnf*), IL-1β (*Il1b*), IL-6 (*Il6*), and MIP-1α (*Ccl3*) (Supplementary Fig. 10). Additionally, elevated protein production of TNF-α, IL-6, and MIP-1α in the culture supernatant was detected following rGGT stimulation (Supplementary Fig. 11). After rGGT stimulation, any production of mature IL-1β production was not detected in the culture supernatant (data not shown); however this may be similar to the known activities of IL-1β and TLR ligands including LPS^28^.

### GGT is not able to activate TLR4-deficient cells

**Figure 2:**
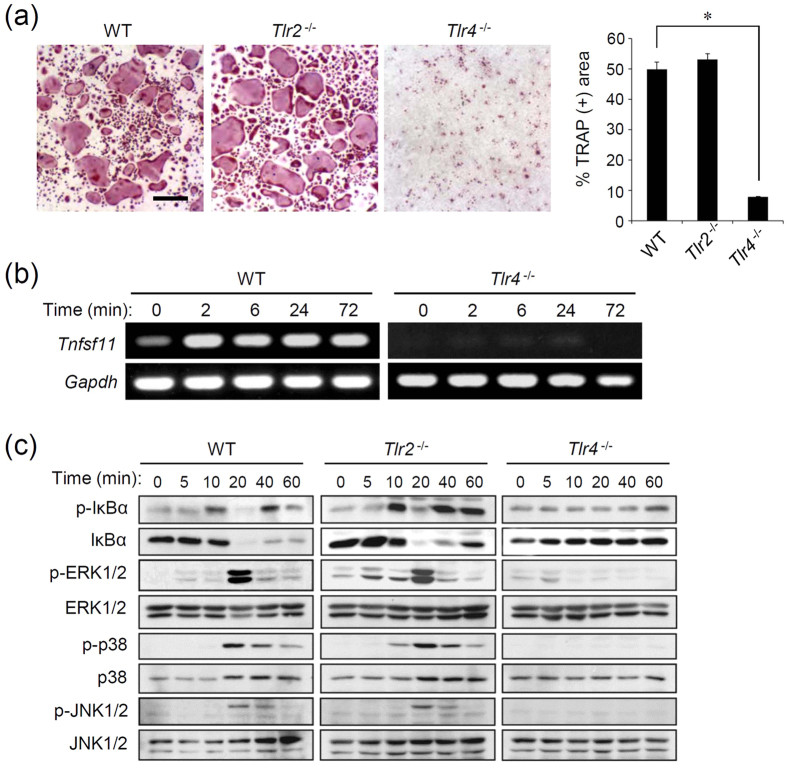
TLR4-deficient cells do not respond to extracellular GGT. (**a**) rGGT-induced formation of OCLs from mouse primary preosteoclasts. Mouse OCPs obtained from WT, *Tlr2*^−/−^, and *Tlr4*^−/−^ mice were differentiated into preosteoclasts using M-CSF and RANKL. Preosteoclasts were stimulated with rGGT (200 ng/ml) in the absence of RANKL for 2 days. Cells were fixed and stained for TRAP. The percentage of the area of TRAP-positive cells was assessed from the microscopic images using the ImageJ software. Data are presented as the mean ± SD (n = 4). Scale: 100 μm. *p < 0.01. (**b**) RT-PCR analysis for RANKL mRNA induction by rGGT stimulation. Mouse primary osteoblasts obtained from WT mice and *Tlr4*^−/−^ mice were stimulated with rGGT (200 ng/ml) for the indicated periods. After extraction of total RNA, expression of *Tnfsf11* (RANKL) and *Gapdh* was assessed. All of the gels were run under the same experimental conditions, and the cropped gel images are shown. (**c**) Immunoblot analysis for NF-κB and MAPK signaling. Phosphorylated IκBα (p-IκBα), IκBα, phosphorylated ERK1/2 (p-ERK1/2), phosphorylated p38 (p-p38), and phosphorylated JNK1/2 (p-JNK1/2) in BMDMs from WT mice, *Tlr2*^−/−^ mice, and *Tlr4*^−/−^ mice stimulated with rGGT (200 ng/ml) were assessed. ERK1/2, p38, and JNK1/2 were used as sample loading controls. All of the blots were under the same experimental conditions, and the cropped images of the blots are shown.

Recent reports have demonstrated that TLRs, especially TLR2 and TLR4, recognize a wide range of endogenous molecules as cognate ligands^11^,^13^,^14^. We therefore aimed to examine whether extracellularly released GGT can be recognized by TLR2 or TLR4 to exert its osteoclastogenic activity. We prepared preosteoclasts from *Tlr2*-deficient (*Tlr2*^−/−^) B6 mice, *Tlr4*-deficient (*Tlr4*^−/−^) B6 mice, and B6 wild-type (WT) mice to investigate osteoclastogenesis. These preosteoclasts were generated with RANKL^low^ treatment of OCPs, and subsequently stimulated with rGGT in the absence of RANKL. WT preosteoclasts and *Tlr2*^−/−^ preosteoclasts were able to differentiate into a significant number of OCLs following rGGT stimulation, while *Tlr4*^−/−^ preosteoclasts failed to differentiate into OCLs (Fig. 2a). Additionally, rGGT stimulation induced the expression of RANKL mRNA (*Tnfsf11*) in osteoblasts obtained from WT mice, but did not induce this expression in cells from *Tlr4*^−/−^ mice (Fig. 2b).

rGGT-induced activation of intracellular signaling events was further examined using BMDMs. Phosphorylation of IκBα and its degradation, both of which are observed upon activation of the transcription factor nuclear factor κB (NF-κB), were observed after rGGT stimulation in WT cells and *Tlr2*^−/−^ cells, but was not observed in *Tlr4*^−/−^ cells (Fig. 2c). Phosphorylation of mitogen-activated protein kinases (ERK1/2, p38 and JNK) were observed in WT cells and *Tlr2*^−/−^ cells and was not observed in *Tlr4*^−/−^ cells (Fig. 2c).

### GGT is recognized by TLR4

**Figure 3:**
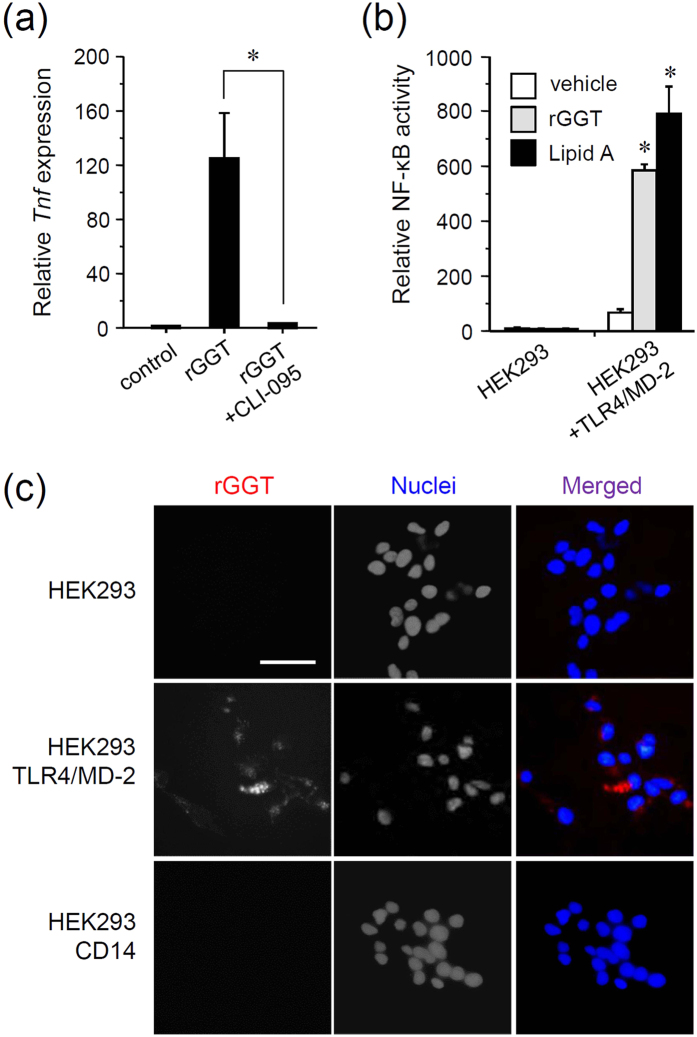
Extracellular GGT is recognized by TLR4. (**a**) Reduction of rGGT-activated *Tnf* expression by the TLR4 inhibitor CLI-095. The bone marrow-derived stromal ST2 cells were preincubated with or without CLI-095 (1 μg/ml) for 6 h, and then stimulated with rGGT (100 ng/ml) for 2 h, followed by RNA extraction and quantitative RT-PCR. Relative expression levels of *Tnf* were determined, and data are expressed as the mean ± SD (n = 4). *p < 0.01. (**b**) rGGT-stimulated NF-κB-dependent transcriptional activity. NF-κB-driven luciferase reporter assay was performed in HEK293 cells and HEK293 cells transfected with TLR4/MD2. Cells were stimulated with rGGT (200 ng/ml) or lipid A (50 ng/ml) for 6 h. Relative NF-κB activity was measured and data are expressed as mean ± SD (n = 3). *p < 0.01. (**c**) Localization of fluorescence-labeled rGGT. HEK293 cells and cells transfected with TLR4/MD2 or with CD14 were treated with AlexaFluor594-labeled rGGT (200 ng/ml) for 6 h. Cells were then fixed and nuclei were counterstained using Hoechst33248. Scale: 50 μm.

We next investigated whether the inhibition of TLR4 affects rGGT-induced response. The TLR4-specific signaling inhibitor CLI-095 (also called TAK-242) was used^29^, and it markedly reduced rGGT-induced *Tnf* expression in ST2 cells (Fig. 3a). Furthermore, we used human embryonic kidney (HEK) 293 cells, which do not express TLR4 and are therefore unresponsive to LPS^30^, to test the effect of transfection of plasmid encoding human TLR4 and its co-receptor MD2. In the control HEK293 cells, it was confirmed that NF-κB-driven promoter activity was not induced by rGGT or lipid A (Fig. 3b). In TLR4/MD2-expressing HEK293 cells, rGGT was able to stimulate NF-κB activity in a similar manner to lipid A. Furthermore, treatment of TLR4/MD2-expressing HEK293 cells with fluorescence-labeled rGGT revealed that GGT was localized partly on the cell surface, and partly inside the cells (Fig. 3c). However, fluorescence-labeled GGT was not observed in the control HEK293 cells and the cells transfected with CD14, another TLR4 co-receptor (Fig. 3c). These results suggest that extracellular GGT is directly recognized by TLR4.

### GGT-induced osteoclastogenesis is dependent on MyD88

**Figure 4:**
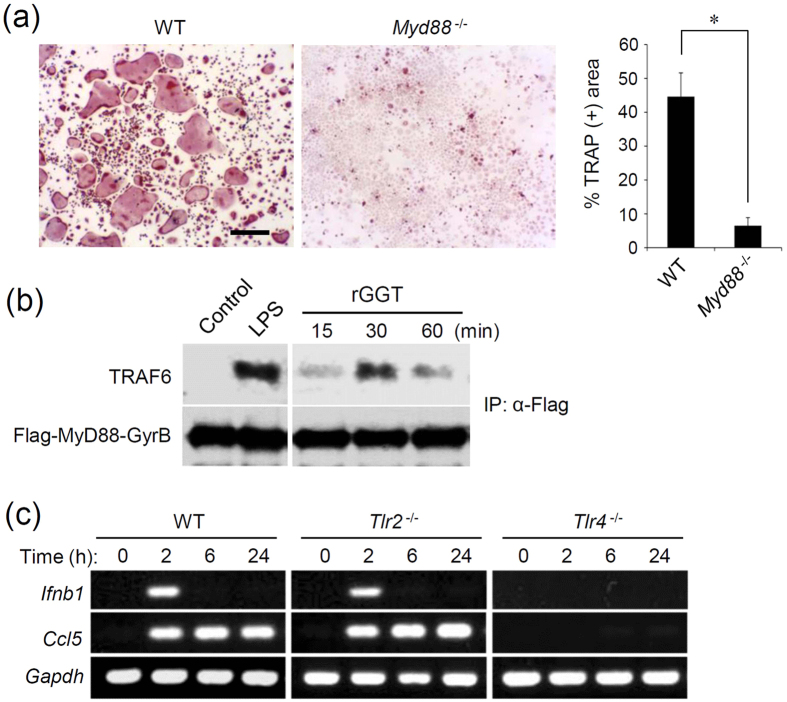
Extracellular GGT requires MyD88-dependent signaling for osteoclastogenesis. (**a**) Formation of OCLs by rGGT stimulation from mouse primary preosteoclasts. Mouse OCPs from WT mice and *Myd88*^−/−^ mice were differentiated into preosteoclasts by treatment with M-CSF and RANKL. Preosteoclasts were stimulated with rGGT (200 ng/ml) in the absence of RANKL for 2 days. Cells were fixed and stained for TRAP. The percentage of the area of TRAP-positive cells was assessed from the microscopic images using the ImageJ software. Data are presented as the mean ± SD (n = 4). Scale: 100 μm. *p < 0.01. (**b**) Immunoprecipitation and immunoblot analysis for MyD88 interaction with TRAF6 to form signaling complex. RAW264.7 cells stably expressing FLAG-MyD88-GyrB were stimulated with 1 μg/ml of rGGT for the indicated periods or with 500 ng/ml LPS for 15 min. Then immunoprecipitation with anti-FLAG M2 agarose (IP: α-FLAG) was carried out with clarified cell lysates, followed by immunoblotting with anti-TRAF6 and anti-FLAG M2 antibodies. The blots have been obtained under the same experimental conditions, and the cropped images of the blots are shown. (**c**) RT-PCR analysis for rGGT induction of mRNA of IFN-β and RANTES. BMDMs from WT mice, *Tlr2*^−/−^ mice, and *Tlr4*^−/−^ mice were stimulated with rGGT (200 ng/ml) for the indicated periods. After extraction of total RNA, expression of *Ifnb1* (IFN-β), *Ccl5* (RANTES), and *Gapdh* was assessed. All of the gels were run under the same experimental conditions, and the cropped gel images are shown.

TLR signaling and RANK-mediated signaling share a downstream TRAF6-mediated pathway^31^. In the TLR signaling, TRAF6-dependent signaling is activated after the recruitment of the adaptor molecule MyD88 to the cytoplasmic domain of TLRs^32^. Of note, MyD88 is reported to be essential for the LPS-triggered TLR4-mediated osteoclastogenesis^33^. Additionally, several DAMPs, including tenascin-C, have been reported to require MyD88 for inducing the production of proinflammatory cytokines^14^. We therefore examined the osteoclastogenic activity of rGGT using preosteoclasts obtained from *Myd88*-deficient (*Myd88*^−/−^) mice. Consistent with the previous results for *Tlr4*^−/−^ preosteoclasts, rGGT-induced osteoclastogenesis was not induced in *Myd88*^−/−^ preosteoclasts (Fig. 4a and Supplementary Fig. 12). rGGT-induced activation of MyD88-dependent signaling was further confirmed as the stimulation-dependent interaction of MyD88 with TRAF6 in RAW264.7 cells (Fig. 4b), which was assessed by the immunoprecipitation of FLAG epitope-tagged MyD88 protein fused to a bacterial DNA gyrase B subunit (GyrB)^34^.

Following the activation of MyD88-dependent signaling at the cell surface, TLR4 subsequently activates MyD88-independent signaling in the early endosomes through the recruitment of another adaptor protein, TIR domain-containing adaptor-inducing interferon-β (TRIF), which is responsible for the production of type I IFN through the activation of TRAF3- and IRF3-dependent signaling^32^. In WT BMDMs and *Tlr2*^−/−^ BMDMs, rGGT stimulation was found to elicit the mRNA expression of IFN-β (*Ifnb1*) and RANTES (*Ccl5*), the typical products of the TRIF-dependent pathway (Fig. 4c). In contrast, the induction of these products was not observed in *Tlr4*^−/−^ cells (Fig. 4c). Thus, our results suggest that extracellular GGT stimulates TLR4, leading to activation of both MyD88-dependent and TRIF-dependent signaling. However, MyD88-dependent signaling seems to preferentially contribute to the GGT-induced osteoclastogenic responses, similar to LPS- or tenascin-C-induced responses^14^,^33^.

### GGT activates TLR4-dependent osteoclastogenesis and inflammation *in vivo*

**Figure 5:**
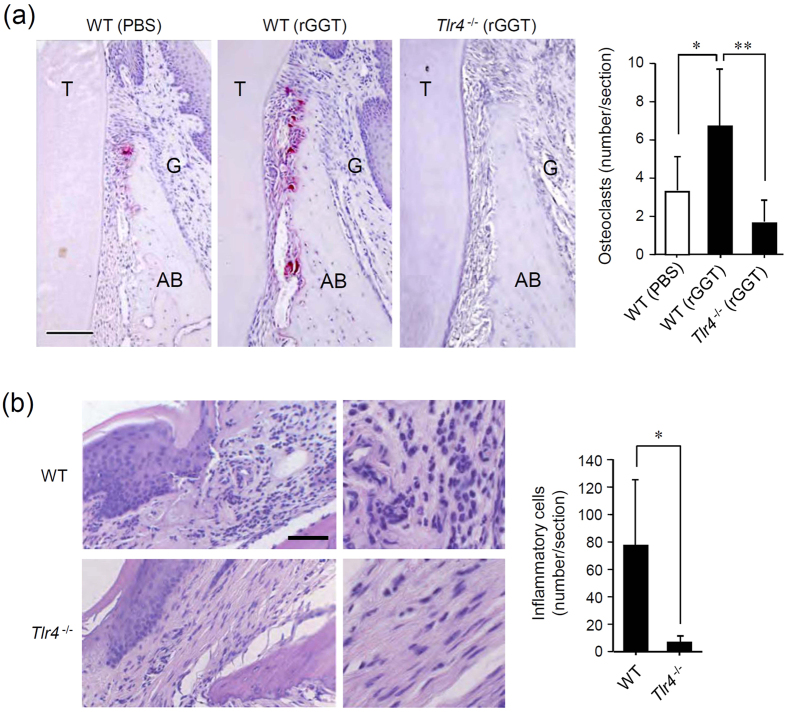
Extracellular GGT induces osteoclastogenesis and acute inflammation *in vivo*. (**a**) Osteoclastogenic effect of rGGT administration. rGGT was administered to the anterior maxillary gingival sulci of WT mice and *Tlr4*^−/−^ mice. AB, alveolar bone; T, tooth; G, gingiva. Scale: 500 μm. The number of osteclasts was assessed by counting TRAP-positive cells within the sections. Data are expressed as the mean ± SD (n = 3). *p < 0.05; **p < 0.01. (**b**) Proinflammatory effect of rGGT administration. rGGT was administered to the labial gingival sulci in the upper incisor of WT mice and *Tlr4*^−/−^ mice. The sections of the gingiva were stained with H&E. The magnified image of the administration site is shown in the right panel. Scale: 100 μm. The number of inflammatory cells was assessed by microscopic counting of hematoxylin-positive polymorphonuclear cells (neutrophils) within the sections. Data are expressed as the mean ± SD (n = 4). *p < 0.05.

We investigated whether extracellular GGT elicits the inflammation-associated osteoclastogenesis *in vivo*. As chronic periodontitis models, rGGT was administered to the gingival sulcus of the upper jaws of WT and *Tlr4*^−/−^ mice. An increased number of TRAP-positive cells indicative of osteoclasts on the alveolar bone surface were generated around the administration site in WT mice, but not in *Tlr4*^−/−^ mice (Fig. 5a). We also investigated the proinflammatory effects of rGGT by administering it into the gingival sulcus to stimulate for 4 h, as a model of acute gingivitis. rGGT caused the infiltration and accumulation of neutrophils in the administration site in WT mice, while no abnormality was observed in *Tlr4*^−/−^ mice (Fig. 5b). Thus, TLR4 was shown to mediate extracellular GGT-induced inflammation-associated osteoclastogenesis and bone resorption.

## Discussion

GGT was previously identified as a lymphocyte-derived osteoclastogenic factor^15^,^19^,^23^. This study provides novel evidence demonstrating TLR4 recognition of extracellular GGT using recombinant proteins and cells from *Tlr4*^−/−^ mice. Our results suggest that extracellular GGT represents a bone metabolism-associated DAMP. The activity of GGT may be similar to LPS because LPS stimulates TLR4 and is known to exert a potent osteoclastogenic activity through TLR4^26^,^35^,^36^. Our results shown here were obtained mainly using preosteoclasts and OCLs since our aim was to investigate the bone metabolism-associated responses; however extracellular GGT seems to be able to activate other types of TLR4-expressing cells as well, including osteoblasts and macrophages, to induce proinflammatory responses in a way that is similar to LPS. Although the mechanism of how extracellular GGT can be released to serve as a TLR4-stimulating DAMP remains unclear, the production and release of GGT from the lymphocytes or inflammatory cells may be stimulated by the inflammation accompanied by oxidative stress. The previous results showed that the transcription of GGT is highly sensitive to oxidative stress^18^, and the elevated humoral GGT levels have been observed in the chronic inflammation-associated diseases for example in the synovial fluids collected from the arthritic joints^23^ and the serum from the inflammatory cardiovascular diseases^20^,^37^. Thus, extracellular GGT may be produced in the inflamed tissues close to the bone in a manner similar to other DAMPs^12^, and therefore, may play a role in promoting the inflammation and inflammation-associated osteoclastogenesis.

GGT is involved in glutathione metabolism and thus helps in maintaining redox homeostasis^18^. GGT knockout mice exhibit high rates of postnatal mortality, which is caused by the high oxidative stress in the lung and by the cellular susceptibility to oxidant injury^38^,^39^,^40^. However, our findings indicate that extracellular GGT has almost the opposite effects through the activation of TLR4-mediated proinflammatory responses. Although the structural basis of GGT-induced TLR4 activation has not been determined, the results of our previous study suggest that it is at least activated in an enzyme-independent fashion^15^. GGT is a single-pass type II membrane glycoprotein, which is initially translated as a propeptide, which is then autocatalytically cleaved into a heterodimeric form comprising a large subunit and a small subunit^41^,^42^. Functionally, the small subunit is the enzymatically active one. We previously reported that a monoclonal antibody, which binds to the large subunit of human GGT1 and never affects enzymatic activity, can efficiently inhibit the osteoclastogenic activity of GGT^15^,^23^. Hence, TLR4 may be recognizing the structure of the large subunit; but additional studies are needed to elucidate the molecular basis of this interaction.

TLR4 is one of the best-characterized members of the TLR family. While it can recognize a wide range of PAMPs and DAMPs^10^,^43^, the best-characterized ligand of TLR4 is LPS. LPS first binds to humoral LBP (LPS-binding protein) and this leads to the disruption of LPS aggregates. The C-terminal domain of LBP subsequently interacts with CD14 localized at the cell surface, which recognizes the carbohydrate chains of LPS to form the monomeric CD14-LPS complex. The LPS acceptor MD2 then binds to the monomeric LPS through recognition of the acylation patterns of the lipid A moiety of LPS. The association of MD2-LPS complex with the ectodomain of TLR4 enables signal transduction through the recruitment of the adaptor molecules^32^,^44^. Although the mechanisms of TLR4-mediated recognition of DAMPs have been remained unclear, it is possible that they are similar to these processes. In the case of extracellular GGT, MD2 seemed to be needed for TLR4-mediated recognition, while CD14 seemed dispensable (Fig. 3). Additional investigations are necessary to determine how TLR4 discriminates extracellular GGT from the membrane GGT. This can be achieved through interaction with another co-receptor, such as CD36, which is known to assist the TLR recognition of serum oxidized LDL and amyloid-β as DAMPs^45^.

GGT is evolutionarily conserved and is found in organisms ranging from bacteria to mammals. Interestingly, GGTs of several pathogenic bacteria were reported to act as virulence factors. GGT of *Helicobacter pylori* converts glutamine into glutamate and ammonia, and glutathione into glutamate and cysteinylglycine, which results in the consumption of glutamine and glutathione, production of ammonia, and generation of reactive oxygen species^46^. These byproducts ultimately induce cell cycle arrest, apoptosis, and necrosis in gastric epithelial cells. Such pathogenic roles of bacterial GGT have also been reported in *Campylobacter jejuni*^47^,^48^. Recently, we found that *H. pylori* GGT can stimulate TLR4, inducing osteoclastogenesis and cytokine production (unpublished data). Given that the TLR4 stimulatory activity of bacterial GGT is associated with pathogenicity, GGT may represent a novel type of PAMP that possesses enzymatic activity. Since GGT production has been identified in several periodontogenic bacterial species including *Aggregatibacter actinomycetemcomitans*^49^, this dual activity may be involved in the pathogenesis of periodontitis accompanied by alveolar bone destruction in a way similar to the host GGT. Of note, we have observed an increase in the enzymatic activity of GGT in the gingival crevicular fluids collected from patients with chronic periodontitis (unpublished data). This suggests that propagated periodontogenic bacteria in the gingival sulcus produce GGT to exacerbate periodontitis through a mechanism similar to endogenous GGT.

Through DAMP recognition, TLR4 is implicated in a diverse range of pathologies, including autoimmune diseases, inflammatory disorders, thrombosis, and cancer^50^. Importantly, the expression of TLR4 is highly elevated in inflamed tissues^51^,^52^,^53^. Furthermore, in addition to GGT, other TLR4-stimulating DAMPs, including tenascin-C^14^ and S100A8/S100A9 proteins^13^, are thought to be expressed in the inflamed tissues. It is therefore likely that several different DAMPs contribute to TLR4 stimulation, and this may lead to the activation of persistent inflammatory signaling. Although it has not been elucidated whether specific DAMPs can preferentially contribute to the development of any diseases, tenascin-C was reported to be involved in the inflammation in arthritic joints^14^. Our previous results, obtained by studying RA patients, animal models of experimental arthritis, and GGT transgenic mice, suggest that GGT is preferentially involved in the diseases characterized by pathological osteoclastogenesis and bone destruction^19^,^23^. This is also supported by the present finding that rGGT induces periodontal disease-like symptoms, including induction of excessive osteoclastogenesis (Fig. 5). It would be of interest to know whether the TLR4-stimulatory activity of GGT is involved in other diseases associated with sterile inflammation. Several studies have indicated that the elevated levels of serum GGT are involved in the pathogenesis of inflammatory cardiovascular diseases^20^,^37^ and TLR4 is also reported to be involved in the development of cardiovascular diseases^54^,^55^.

DAMPs can potentially be used as clinical markers for disease progression and severity, but further studies are needed in order to establish their detailed roles and specificity. Additionally, targeting DAMPs and their receptors has been regarded as a useful strategy in the treatment of severe inflammatory conditions^11^. Our results provide important evidence supporting the potential of the use of TLR4 agonist for the treatment of inflammatory diseases accompanied by bone destruction, which are aggravated by TLR4-stimulating DAMPs, including extracellular GGT.

## Materials and Methods

### Reagents and plasmids

Recombinant proteins of mouse RANKL, M-CSF and TNF-α, and antibody to mouse TNF-α (AF-410) were purchased from R&D Systems. The TNF-α neutralizing activity of the antibody is assured by the supplier. Recombinant osteoprotegerin was described previously^15^. *Escherichia coli* LPS 0111:B4 (L3024) and PMB sulfate salt (P4932) were purchased from Sigma-Aldrich. The synthetic product of *E. coli* lipid A (compound 506) was obtained from Peptide Institute (Osaka, Japan). The TLR4 signaling inhibitor CLI-095 was obtained from InvivoGen. Plasmid encoding human CD14 was a kind gift from K. Shibata (Hokkaido University, Sapporo, Japan).

### Preparation of rGGT

rGGT was prepared using baculoviral particles encoding whole human GGT1 protein and *Spodoptera frugiperda*-derived Sf2 cells as described previously^23^. LPS concentration in rGGT solution was determined by *Limulus* amoebocyte lysate assay, using Endospacy ES-50M Set (Seikagaku Co., Tokyo, Japan). Pretreatment of rGGT (10 μg) or lipid A (10 μg) with proteinase K (100 μg/ml) or with PMB (100 μg/ml) were performed at 37 °C for 2 h. Fluorescence-labeled rGGT was obtained using AlexaFluor594 Microscale Protein Labeling Kit (Life Technologies) according to the manufacturer’s instructions.

### Mice

All animal studies were approved by the Animal Experimental Committees of the National Center for Geriatrics and Gerontology (NCGG) and were carried out in accordance with approved guidelines. B6 background mutant mice (*Tlr2*^−/−^, *Tlr4*^−/−^ and *Myd88*^−/−^ mice) were obtained from Oriental Bio Service (Kyoto, Japan). Control B6JJcl mice were obtained from CLEA Japan (Tokyo, Japan). All mice were maintained in the animal facilities of NCGG and were housed in a conventional animal room (temp, 22 ± 2 °C; humidity, 50%; light/dark cycle, 12 h) with free access to food and water. Mice used in the same experiment were matched by age (7–8 weeks) and sex (male), and they were sacrificed by cervical dislocation. All efforts were made to minimize animal suffering.

### Cells

Mouse bone marrow hematopoietic cells were collected from the femur of male B6 mice as described previously^23^. Mouse primary osteoblasts were isolated from calvariae of newborn B6 mice as described previously^56^. Human OCPs were prepared using the Poietics Human Osteoclast Precursor Cell System (Lonza), according to the manufacturer’s instructions. Mouse BMDMs were prepared from bone marrow hematopoietic cells by washing them once and resuspending them in α-MEM supplemented with 10% fetal bovine serum (FBS), 50 ng/ml M-CSF, 100 mM sodium pyruvate, 0.1 mM non-essential amino acids, 2 mM L-glutamine, 100 U/ml penicillin, 100 mg/ml streptomycin, and 50 μg/ml gentamycin. Highly adherent cells were allowed to adhere overnight in 100-mm diameter culture dishes and then removed. Non-adherent cells were collected and plated in the culture dishes and incubated for 3 days, in order to obtain adherent macrophages. RAW264.7 cells, ST2 cells and human embryonic kidney (HEK) 293 cells were obtained from RIKEN Cell Bank (Kanagawa, Japan), and maintained in α-MEM containing 10% FBS and DMEM containing 10% FBS, respectively. HEK293 cells with the stable expression of human TLR4/MD2 were obtained from InvivoGen. The preparation of RAW264.7 cells stably expressing FLAG epitope-tagged MyD88-GyrB was described previously^57^. All cells were cultivated at 37 °C in an atmosphere with 5% CO_2_.

### *In vitro* osteoclastogenesis assay

Analysis of *in vitro* osteoclastogenesis was performed as described previously^23^. Briefly, bone marrow hematopoietic cells were plated in 100-mm diameter culture dishes and incubated with media containing 50 ng/ml of M-CSF. After 4 days, the adherent BMDMs were collected and used as OCPs to be plated in 96-well plates at 1 × 10^4^ cells/well in α-MEM containing 10% FBS in the presence of 50 ng/ml M-CSF and 20 ng/ml RANKL for 5 days to obtain mononuclear preosteoclasts. After medium change, preosteoclasts were stimulated with 200 ng/ml rhGGT or 50 ng/ml lipid A in the absence of RANKL for 2 days to induce osteoclastogenesis. For the generation of RAW264.7 preosteoclasts, RAW264.7 cells were plated in a 96-well plate at a density of 4 × 10^3^ cells/well and stimulated with RANKL^low^ for 3 days. After medium change, these cells were stimulated with 200 ng/ml rGGT or 50 ng/ml lipid A for 24 h. For the inhibition study, OCPs were preincubated with osteoprotegerin (100 ng/ml) or anti-mouse TNF-α (5 μg/ml) 1 h before rGGT stimulation. To confirm multinucleated OCL formation, the cultured cells were fixed with PBS containing 10% formalin for 3 min, and stained for the osteoclast marker enzyme TRAP. The TRAP-positive cells with more than three nuclei were considered and their number was determined using light microscopy. The percentage of TRAP-positive area was analyzed from the RGB-splitted microscopic images using the ImageJ software (version 1.49; National Institutes of Health, Bethesda, Maryland).

### Microscopic analysis

rGGT localization was investigated by culturing the cells on poly-L-lysine coated culture slides (Becton Dickinson), and treating them with Alexa 546-labeled rGGT for 2 h, followed by fixation in 4% paraformaldehyde. Cell nuclei were stained with Hoechst33258 (Molecular Probes). For immunofluorescent staining of OCLs, RAW264.7-derived OCLs were cultured on Lab-Tek 8-well permanox chamber slides (Thermo Fisher Scientific). Cells were fixed in PBS with 10% formalin and then treated with 0.1% Triton X-100 in PBS. They were subsequently stained with rhodamine-phalloidin to detect filamentous actin and rabbit anti-cathepsin K antibody, followed by the staining with Alexa488-conjugated anti-rabbit IgG antibody (Life Technologies). Stained cells were embedded in ProlongGold Antifade reagent (Life Technologies). Images were obtained using an LSM 710 confocal microscope system (Carl Zeiss).

### Pit formation assay

The bone resorbing activity of OCLs was measured using RAW264.7 preosteoclasts cultured on BD BioCoat Osteologic 12.7 mm Discs (BD Biosciences) placed in 24-well plates. Cells (2.5 × 10^4^ cells/well) were pretreated with RANKL^low^ for 3 days and then treated with 50 ng/ml RANKL or with 200 ng/ml rGGT for 2 days. After cultivation, the discs were rinsed with distilled water and left for 5 min in 6% sodium hypochlorite to remove all of the attached cells. The disks were then washed with distilled water and air-dried. Pits formed on the discs were observed using phase-contrast microscopy.

### Reverse transcription-polymerase chain reaction (RT-PCR)

Total RNA was extracted from cultured cells using RNeasy mini kit (QIAGEN), and reverse-transcribed using Ready-To-Go You-Prime First-Strand Beads (GE Healthcare) according to the manufacturer’s instruction. cDNA was amplified using Taq DNA polymerase (TaKaRa) and the primer sets shown in Supplementary Table 1. The PCR products were separated by electrophoresis on a 1.2% agarose gel and visualized using ethidium bromide staining under UV light. Quantitative RT-PCR for the determination of *Tnf* expression in ST2 cells and *Il1b* expression in RAW264.7 cells was performed by referring to the method described previously^57^,^58^.

### Determination of cytokine concentrations by enzyme-linked immunosorbent assay (ELISA)

RAW264.7 cells (5 × 10^4^/well) were cultured in 96-well plates and stimulated with 200 ng/ml of rGGT for 12 h. The concentrations of MIP1α (MMA00), TNF-α (MTA00B), IL-6 (M6000B) and mature IL-1β (MLB00C) in the culture supernatants were determined by Quantikine ELISA Kit (R&D Systems) according to the manufacturer’s instructions.

### Immunoblotting

Mouse BMDM cell lysates were obtained using a lysis buffer containing 10 mM Tris-HCl (pH 7.8), 150 mM NaCl, 1 mM EDTA, 1% Nonidet P-40, 5 mM sodium orthovanadate, 5 mM NaF, and a protease inhibitor mixture (Complete; Roche Diagnostics). Cell lysates were centrifuged, and the supernatants were boiled and subjected to SDS-polyacrylamide gel electrophoresis (10% gel) under te reducing conditions. Immunoblotting was performed using phosphorylation-specific primary antibodies to IκBα (2859), p38 (4631), JNK (4671), ERK (4376), antibodies to p38 (9212), JNK (9258), ERK1/2 (9102), and IκBα (9242) obtained from Cell Signaling Technology, and horseradish peroxidase-conjugated anti-IgG secondary antibodies. Immunoreactive bands were detected using ECL Plus reagent (GE Healthcare).

### Luciferase assay for NF-κB activity

Luciferase reporter gene assay for the determination of NF-κB-driven promoter activity was performed as described previously^59^. Dual luciferase activities were measured using Dual-Luciferase reporter assay system (Promega) and a Berthold luminometer (Berthold, Bundoora, Australia) according to the manufacturer’s instructions.

### Immunoprecipitation

The immunoprecipitation of FLAG-tagged MyD88-GyrB using the clarified lysates of RAW264.7 cells was performed according to the method described previously^57^.

### *In vivo* osteoclastogenesis

To assess the osteoclastogenic activity of GGT *in vivo*, 2 μl of 50 μg/ml rGGT was administered to the buccal gingival sulci of the upper molar of anesthetized mice using a micropipette. The process was done 6 times, once every 10 min. After 3 days of administration, the mice were sacrificed, the upper jaws were resected en bloc, and the specimens were fixed in 4% paraformaldehyde in PBS for 12 h at 4 °C. The hard tissues were decalcified in 10% EDTA for 10 days. All samples were embedded in Tissue-tech wax (Sakura Fine Tech, Tokyo, Japan) and 7-μm thick sections were obtained. Sections were stained for osteoclast-specific TRAP activity and counterstained with Mayer’s hematoxylin, as described previously^23^. TRAP-positive cells in each section were counted under a light microscope. Routine hematoxylin and eosin (H&E) staining was performed as well.

### Acute gingivitis induction by administration of rGGT

Acute gingivitis was induced by the administration of 20 μl of 250 μg/ml rGGT into the labial gingival sulci in the upper incisor of anesthetized mice, using a micropipette. After 4 h of administration, mice were sacrificed, and their upper jaws were decalcified, paraffin-embedded, and serially sectioned. The sections were stained with H&E. Hematoxylin-positive polymorphonuclear cells (neutrophils) in each section were considered as inflammatory cells while counting under a light microscope.

### Statistical analysis

Data were expressed as the mean ± standard deviation (SD). P values were calculated using unpaired Student’s *t*-test, and values less than 0.05 or 0.01 were considered significant.

## Additional Information

**How to cite this article**: Moriwaki, S. *et al.* γ-Glutamyltranspeptidase is an endogenous activator of Toll-like receptor 4-mediated osteoclastogenesis. *Sci. Rep.* **6**, 35930; doi: 10.1038/srep35930 (2016).

## Supplementary Material

Supplementary Information
