# Supplementary material for: γ-Glutamyltranspeptidase is an endogenous activator of Toll-like receptor 4-mediated osteoclastogenesis
Source: Sci Rep. 2016 Oct 24;6:35930. doi: 10.1038/srep35930 (PMC5075938; doi:10.1038/srep35930)
Supplement: Supplementary Information [file srep35930-s1.pdf]

Supplementary Information

γ-Glutamyltranspeptidase is an endogenous activator of Toll-like receptor 4-mediated osteoclastogenesis

Sawako Moriwaki[1,†,*], Takeshi Into[2,*], Keiko Suzuki[3,‡], Mutsumi Miyauchi[4], Takashi Takata[4], Keigo Shibayama[5] & Shumpei Niida[1**]

[1]Biobank, Medical Genome Center, National Center for Geriatrics and Gerontology (NCGG), Obu 474-8522, Japan.

[2]Department of Oral Microbiology, Division of Oral Infections and Health Sciences, Asahi University School of Dentistry, Mizuho 501-0296, Japan.

[3]Department of Pharmacology, School of Dentistry, Showa University, Tokyo 142-8555, Japan.

[4]Department of Oral and Maxillofacial Pathology, Institute of Biomedical & Health Sciences, Hiroshima University, Hiroshima 734-8522, Japan.

[5]Department of Bacteriology II, National Institute of Infectious Diseases, Tokyo 208-0011, Japan.

[†]Present address: Biobank of National Cerebral and Cardiovascular Center, Osaka Japan.

[‡]Present address: Department of Pharmacology, School of Dentistry, Ohu University, Fukushima, Japan.

*These authors contributed equally to this work.

**Correspondence should be addressed to S.N. (sniida@ncgg.go.jp)

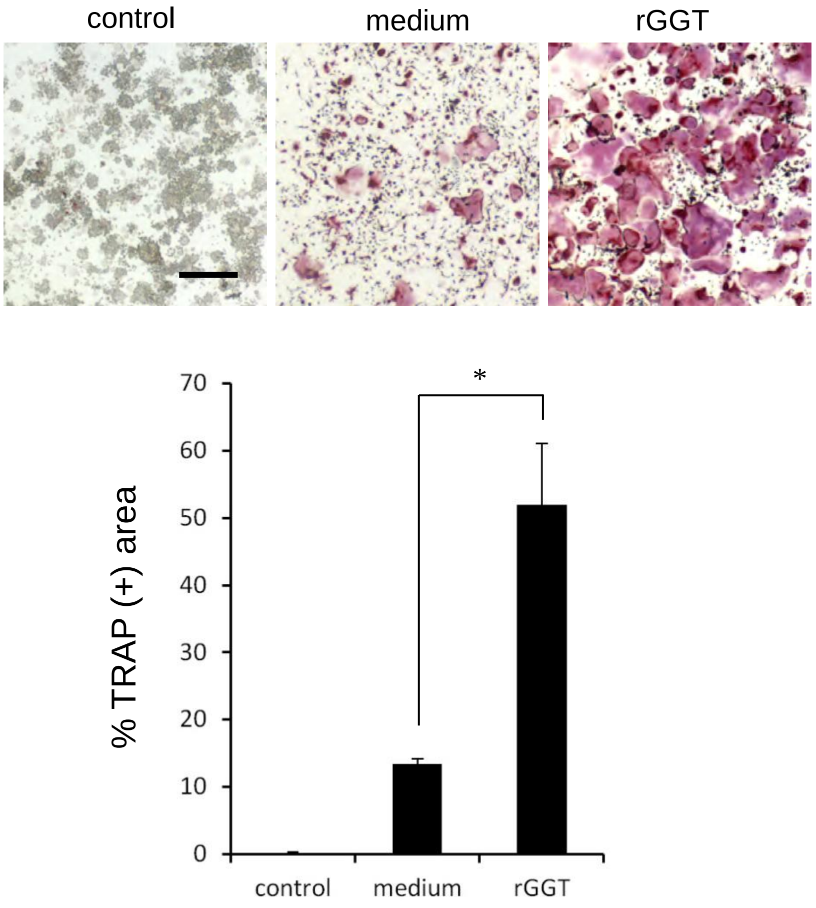

Supplementary Figure 1. rGGT-induced formation of osteoclasts from mouse primary OCPs. Mouse bone marrow hematopoietic cells were differentiated into OCPs by treatment with M-CSF (panel of control). OCPs were then stimulated with M-CSF and $RANKL^{low}$ for 5 days to obtain preosteoclasts (panel of medium). Preosteoclasts were stimulated with rGGT (200 ng/ml) in the absence of RANKL for an additional 2 days (panel of rGGT). Cells were fixed and stained for TRAP. Microscopic images were obtained and the percentage of the area of TRAP-positive cells was analyzed using the ImageJ software. Results are shown as the mean ± SD (n=4). Scale: 100 μm. *, $p < 0.01$.

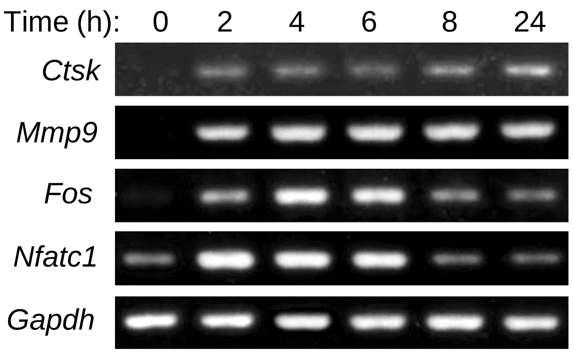

Supplementary Figure 2. RT-PCR analysis for rGGT induction of genes characteristic of osteoclasts.

Mouse primary OCP-derived preosteoclasts were stimulated with rGGT (200 ng/ml) in the absence of RANKL during the indicated periods. After extraction of total RNA, the expression of osteoclastogenic genes *Ctsk*, *Mmp9*, *Fos*, and *Nfatc1* was assessed. All of the gels were run under the same experimental conditions, and the cropped gel images are shown.

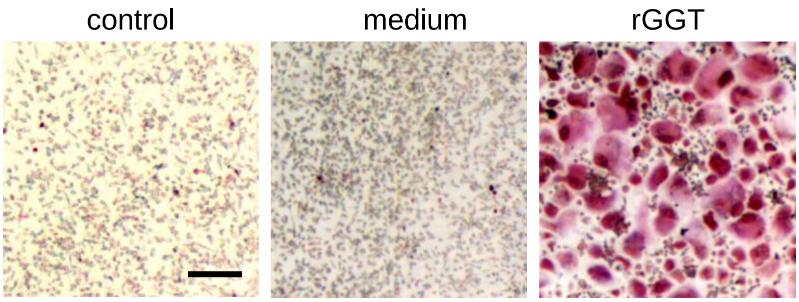

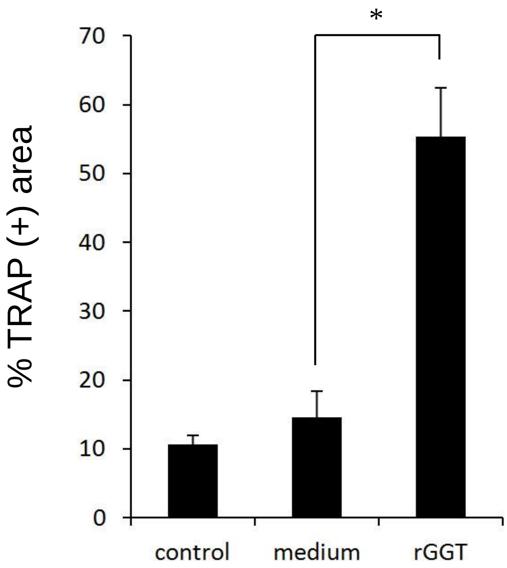

Supplementary Figure 3. Extracellular GGT induces osteoclastogenesis from human primary OCPs.

Human primary OCPs were prepared using the Poietics Human Osteoclast Precursor Cell System (Lonza), according to manufacturer's instructions, and seeded in 96-well plates (1×10^4 cell/wells). OCPs were cultured with optimal RANKL in the presence of M-CSF for 5 days (panel of control). Cells were then stimulated with rGGT (300 ng/ml; panel: rGGT) or without rGGT (panel of medium) in the absence of RANKL for additional 2 days. Cells were then fixed and stained for TRAP. Microscopic images were obtained and the percentage of the area of TRAP-positive cells was analyzed using the ImageJ software. Results are shown as the mean ± SD (n=4). Scale: 100 μm. *, $p < 0.01$.

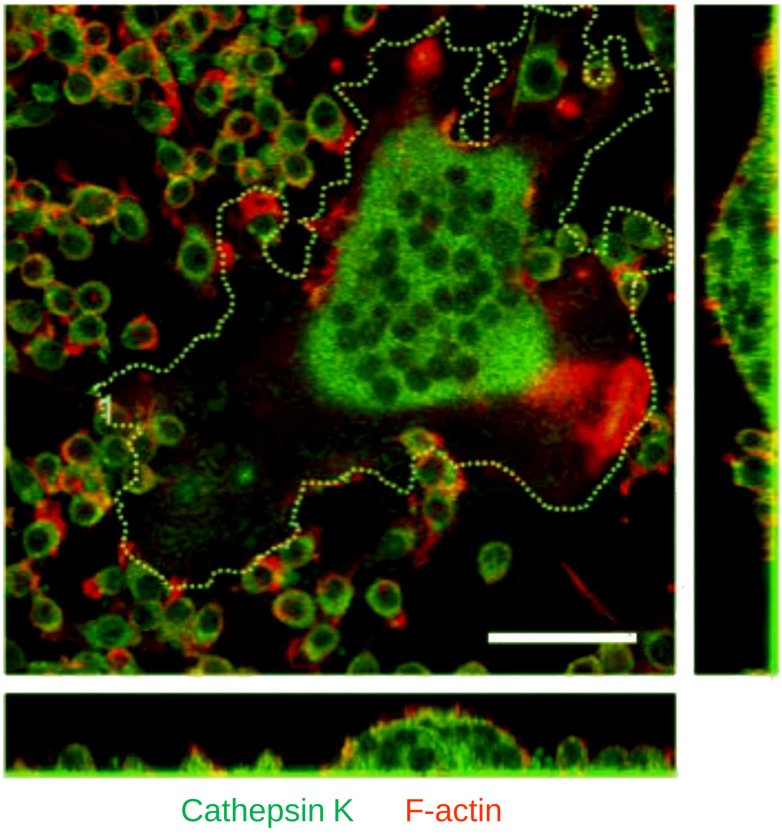

Supplementary Figure 4. Confocal microscopic image of an rGGT-induced osteoclast-like cell. RAW264.7-derived preosteoclasts were stimulated with rGGT (200 ng/ml) in the absence of RANKL for 24 h. Cells were fixed and fluorescently stained for cathepsin K (green) and F-actin (red). Dotted lines indicate the cell shape. The images of cellular depth are also shown. Scale: 50 μm.

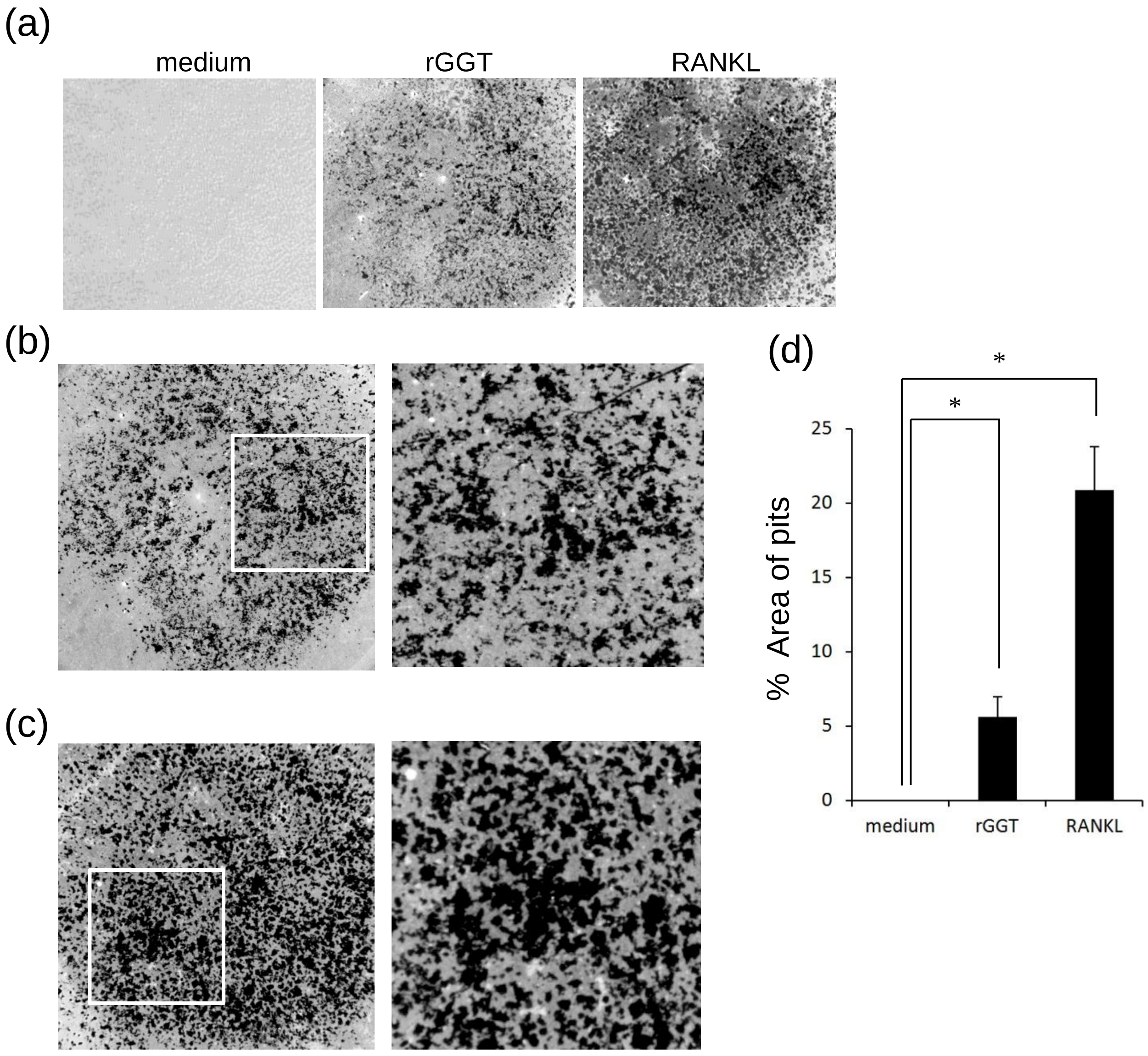

Supplementary Figure 5. Pit formation by rGGT-induced osteoclast-like cells *in vitro*.
(a) RANKL^{low}-treated RAW264.7 preosteoclasts were stimulated with rGGT (200 ng/ml) in the absence of RANKL (panel of rGGT) or were untreated (panel of medium) on the calcified matrix-coated disks for 24 h. Cells stimulated with RANKL (50 ng/ml) were also prepared as a positive control (panel of RANKL). Black dots indicate resorption pits formed on the disks.
(b) Another image of ‘rGGT’ (left panel) and its magnified image (right panel).
(c) Another image of ‘RANKL’ (left panel) and its magnified image (right panel).
(d) The percentages of the area of pits analyzed from three microscopic images using the ImageJ software. Results are shown as the mean ± SD (n=3). *, $p < 0.01$.

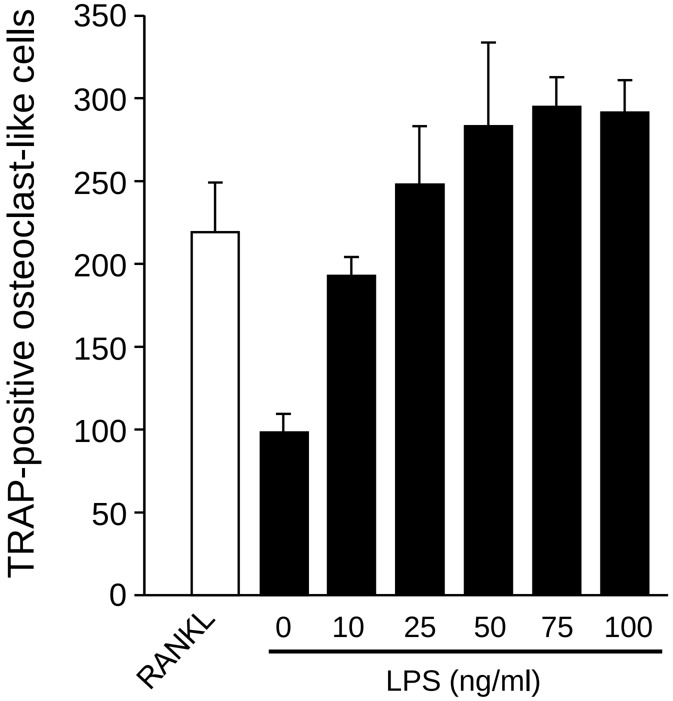

Supplementary Figure 6. Effect of LPS stimulation on osteoclastogenesis.
RAW264.7 cells were differentiated into preosteoclasts with RANKL^{low} in 96-well plates for 3 days. Preosteoclasts were stimulated with the indicated concentrations of *E. coli* LPS (black bars) or with RANKL (100 ng/ml; white bar) for 24 h. Cells were then fixed and stained for TRAP and the numbers of TRAP-positive multinucleated osteoclast-like cells were counted. Results shown are the mean ± SD.

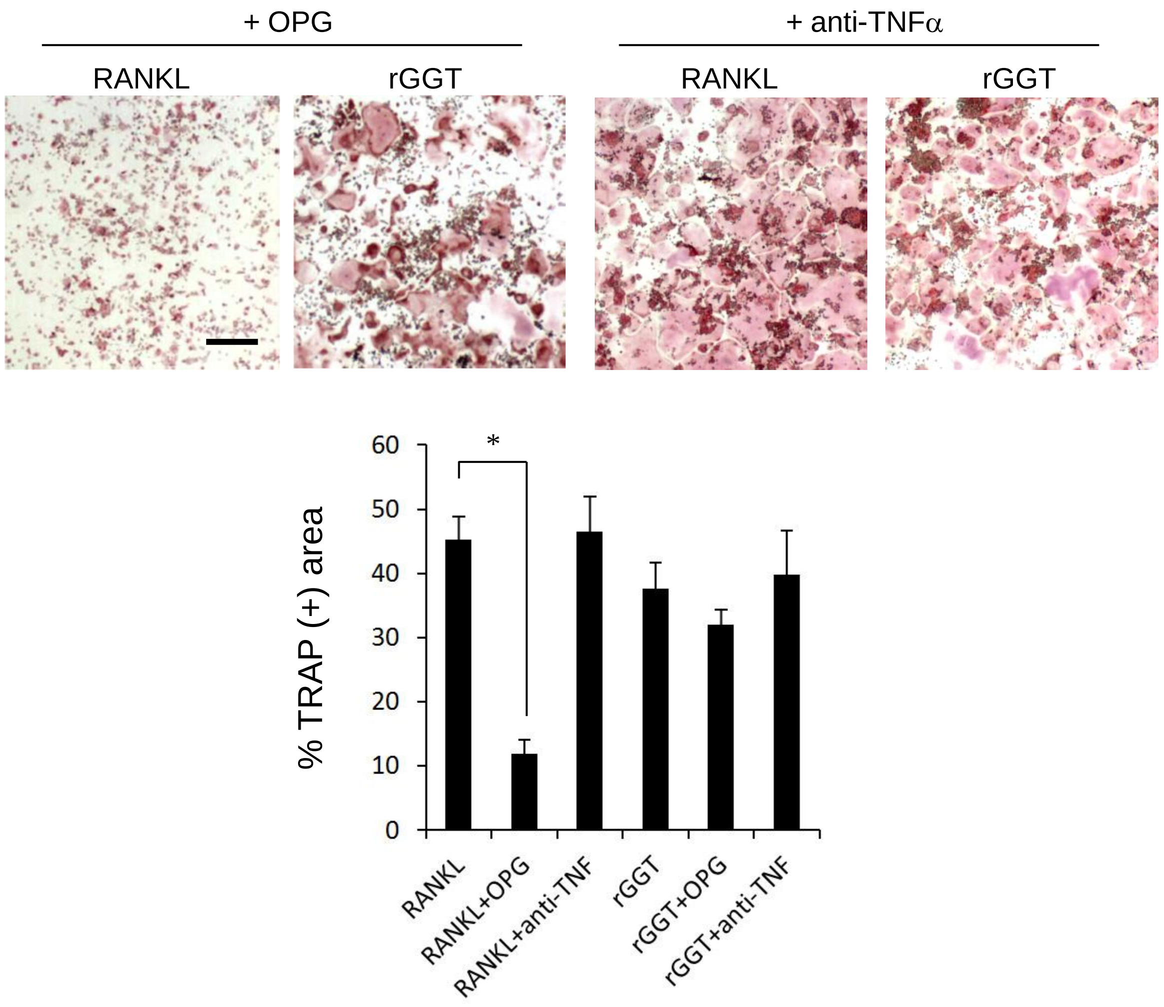

Supplementary Figure 7. Osteoprotegerin or neutralization of TNF-α does not affect rGGT-induced osteoclastogenesis.

RAW264.7 cells were differentiated into preosteoclasts with $RANKL^{low}$ for 3 days. These cells were subsequently stimulated with RANKL (100 ng/ml; left panel) or rGGT (200 ng/ml; right panel) in the presence of osteoprotegerin (OPG; 100 ng/ml) or TNF-α-neutralizing antibody (5 μg/ml) for 24 h. Cells were fixed and stained for TRAP. The panels of 'RANKL' and 'rGGT' are shown in Fig. 1. Microscopic images were obtained and the percentage of the area of TRAP-positive cells was analyzed using the ImageJ software. Results are shown as the mean ± SD (n=4). Scale: 100 μm. *, $p < 0.01$.

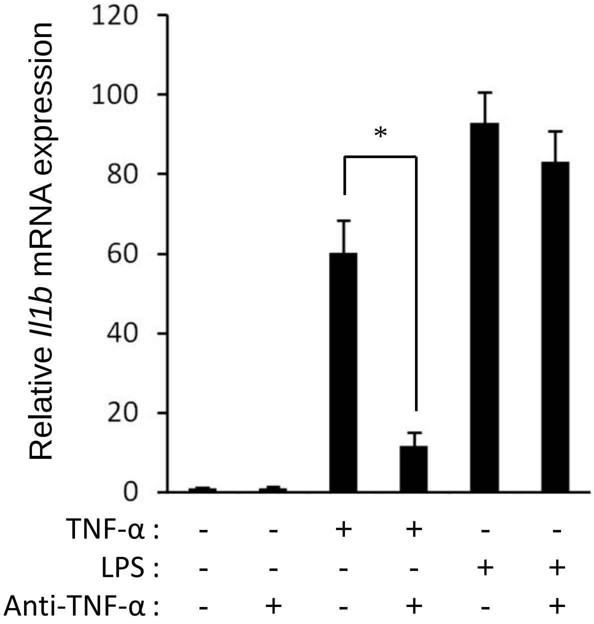

Supplementary Figure 8. Control experiment to demonstrate effectiveness of anti-TNF-α antibody.

RAW264.7 cells (2.5×10^5 cells) were cultured in the presence or absence of TNF-α-neutralizing antibody (5 µg/ml) for 1 h, and then stimulated with recombinant mouse TNF-α (1 ng/ml) or with LPS (100 ng/ml) for 3 h. After extraction of total RNA from the cells, *Il1b* mRNA induction was determined by quantitative RT-PCR analysis. Results, expressed as relative *Il1b* mRNA expression, are shown as the mean ± SD (n=3). *, $p < 0.01$.

The antibody efficiently blocked the activity of TNF-α, but it hardly affected the activity of LPS.

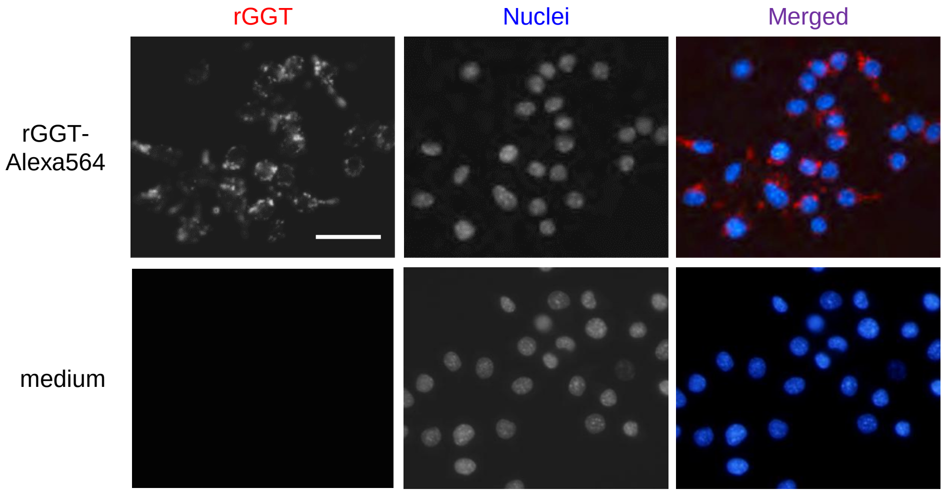

Supplementary Figure 9. Localization of fluorescence-labeled rGGT.
RAW264.7 cells were treated with AlexaFluor594-labeled rGGT (200 ng/ml) for 6 h and fluorescent microscopy images were obtained. Cells were fixed and nuclei were counterstained using Hoechst33248. Scale: 50 µm.

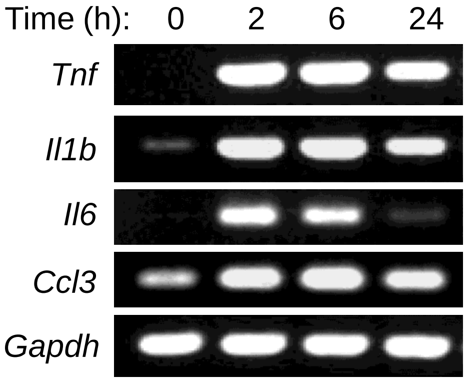

Supplementary Figure 10. RT-PCR analysis for rGGT induction of proinflammatory cytokine genes.
Mouse BMDMs were stimulated with rGGT (200 ng/ml) during the indicated periods. After total RNA extraction, the expression of *Ccl3* (MIP1α), *Tnf* (TNF-α), *Il6* (IL-6), and *Il1b* (IL-1β), was assessed. All of the gels were run under the same experimental conditions, and the cropped gel images are shown. The image of *Gapdh* is the same as the image shown in Fig. 4C.

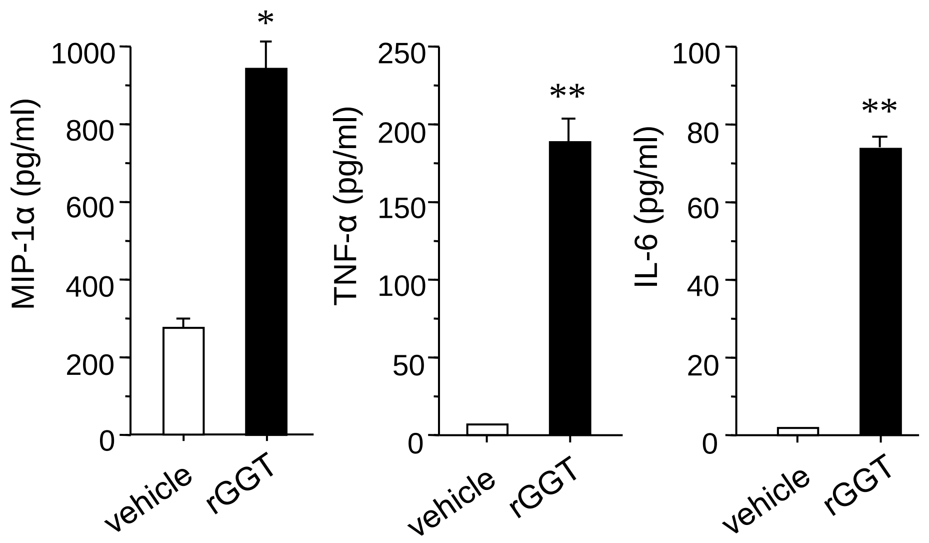

Supplementary Figure 11. ELISA for proinflammatory responses by extracellular GGT stimulation.
RAW264.7 cells were stimulated with rGGT (200 ng/ml) for 12 h. The concentrations of MIP1α, TNF-α, and IL-6 in the culture supernatants were measured. Data are presented as the mean ± SD (n=3). *, $p < 0.05$; **, $p < 0.01$.

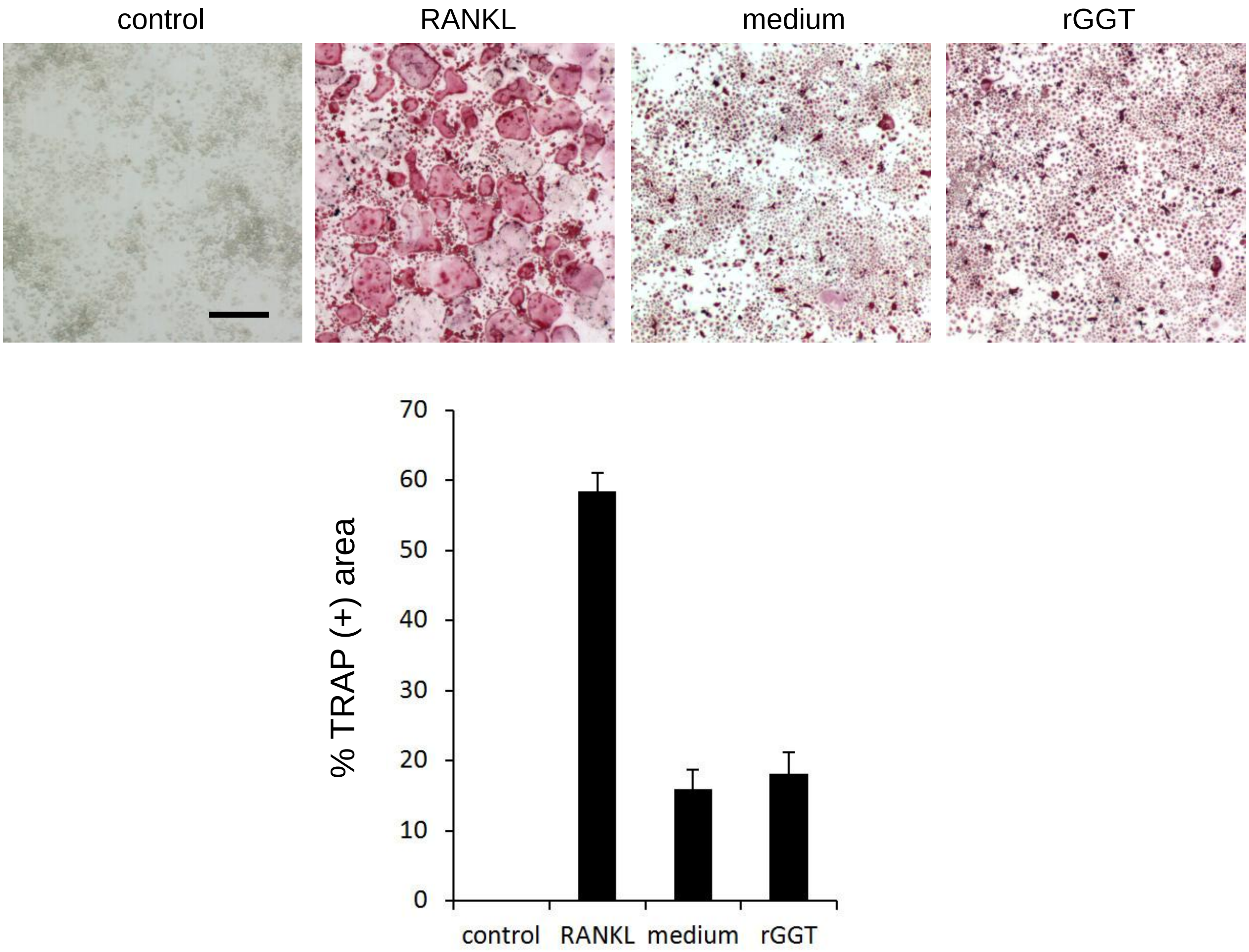

Supplementary Figure 12. ***In vitro*** **osteoclastogenesis in** ***Myd88$^{-/-}$*** **OCPs.**
Mouse bone marrow hematopoietic cells obtained from *Myd88$^{-/-}$* mice were differentiated into OCPs by treatment with M-CSF for 4 days (panel: control). OCPs were subsequently stimulated with RANKL^{low} in the presence of M-CSF for 5 days to obtain preosteoclasts. Preosteoclasts were left untreated (panel of medium) or stimulated with rGGT (250 ng/ml) in the absence of RANKL (panel of rGGT) or stimulated with RANKL (100 ng/ml; panel of RANKL) for additional 2 days. Cells were fixed and stained for TRAP. Microscopic images were obtained and the percentage of the area of TRAP-positive cells was analyzed using the ImageJ software. Results are shown as the mean ± SD (n=4). Scale: 100 μm.

Supplementary Table 1. Primer sets used in RT-PCR analysis.

Gene	Sequence (5’ → 3’)
Ctsk	Forward: GGGAGACATGACCAGTGAAG
	Reverse: TCTCTTCAGGGCTTTCTCGT
Mmp9	Forward: CCAGGATAAACTGTATGGCT
	Reverse: CAGGAACTTCCAGTACCAAC
Fos	Forward: TCTAGTGCCAACTTTATCCCC
	Reverse: GAAGTCATCAAAGGGTTCCTG
Nfatc1	Forward: TCGGTAACACCACCCAGTAT
	Reverse: ACAGCTGTAGCGTGAGAGGT
Ccl3	Forward: ACACTCTGCAACCAAGTCTTC
	Reverse: GACCCAGGTCTCTTTGGAGT
Tnf	Forward: GCCTCTTCTCATTCCTGCTT
	Reverse: GTACTTGGGCAGATTGACCT
Il6	Forward: TTGCCTTCTTGGGACTGATG
	Reverse: CTGAAGGACTCTGGCTTTGT
Il1b	Forward: GCATCCAGCTTCAAATCTCAC
	Reverse: TCCTGACCACTGTTGTTTCC
Ifnb1	Forward: CACAGCCCTCTCCATCAACT
	Reverse: AGTTGAGGACATCTCCCACG
Ccl5	Forward: CCTCACCATCATCCTCACTG
	Reverse: CCACTTCTTCTCTGGGTTGG
Tnfrsf11	Forward: ATCAGAAGACAGCACTCACT
	Reverse: TCATCAGGTTATGAGAACTTGG
Gapdh	Forward: ACCACAGTCCATGCCATCAC
	Reverse: TCCACCACCCTGTTGCTGTA
